# Oxaloacetate as a Holy Grail Adjunctive Treatment in Gliomas: A Revisit to Metabolic Pathway

**DOI:** 10.7759/cureus.48821

**Published:** 2023-11-15

**Authors:** Abdul Samad, Rajaram Samant, K Venkateshwara Rao, Vyom Bhargava, Shahid I Sadique, Rohit Yadav

**Affiliations:** 1 Department of Medical Affairs, Celagenex Research (India) Private Limited, Thane, IND; 2 Department of Medicine, Celagenex Research (India) Private Limited, Thane, IND; 3 Department of Neurosurgery, Basavatarakam Indo American Cancer Hospital and Research Institute, Hyderabad, IND; 4 Department of Neurosurgery, HMC Hospital, Ludhiana, IND; 5 Department of Neurosurgery, Institute of Post-Graduate Medical Education and Research (IPGMER) and Seth Sukhlal Karnani Memorial (SSKM) Hospital, Kolkata, IND

**Keywords:** glutamate excitotoxicity, alter warburg effect, glioma, adjunctive therapy, oxaloacetate

## Abstract

India experiences a significant amount of morbidity and mortality due to gliomas particularly glioblastoma multiforme (GBM), which ranks among the worst cancers. Oxaloacetate (OAA) is a human keto acid that is central to cellular metabolism; it has been recognized by the US FDA for use in GBM patients, triggering a review to revisit the cellular mechanism of its therapeutic action. Various cellular and molecular studies have proposed that instead of fueling the tricarboxylic acid (TCA) cycle and oxidative phosphorylation (OXPHOS), gliomas prefer to use glycolysis (the Warburg effect) to fuel macromolecules for the synthesis of nucleotides, fatty acids, and amino acids for the accelerated mitosis. A study found that oxaloacetate (OAA) inhibits human lactate dehydrogenase A (LDHA) in cancer cells, reversing the Warburg effect. Studies revealed that OAA supplementation reduced Warburg glycolysis, improved neuronal cell bioenergetics, and triggered brain mitochondrial biogenesis, thereby enhancing the efficacy of standard treatment. Similarly, OAA has been found in preclinical investigations to be able to decrease tumor development and survival rates by blocking the conversion of glutamine to alpha-ketoglutarate (alpha-KG) in the TCA cycle and lowering nicotinamide adenine dinucleotide phosphate (NADPH) levels. OAA is a safe adjuvant that has the potential to be an effective therapy in gliomas when combined with temozolomide (TMZ) chemotherapy and routine surgery.

## Introduction and background

Cancer is a significant public health issue on a global scale [1]. As per the GLOBOCAN 2020 statistics, there were 308,102 newly reported cases of brain and other central nervous system cancers, with a corresponding 251,329 fatalities [2]. The majority of malignant brain tumors (80%) are gliomas [3], which have been categorized and graded based on histological characteristics [4]. According to data from the hospital-based cancer registry collected by the Indian Council of Medical Research in 2021, brain tumors represented 1.6% of all cancerous growths [5]. This study also revealed a high incidence of glioma in various Indian cities, with males at 5.8% and females at 21.8%. Among all gliomas, glioblastoma multiforme (GBM) is a deadly, complex, and treatment-resistant tumor. Only 5% of patients survive five years after diagnosis. The current standard of care involves surgical removal, radiation, and temozolomide (TMZ) chemotherapy. The median survival time is 14.6 months, and the two-year survival rate is 26.5%. However, most GBMs return to the original site, and there is no accepted standard of care for recurrent GBMs [6-8].

TMZ, a standard chemotherapy treatment for malignant gliomas, has shown limited efficacy due to inherent and acquired drug resistance [9]. Improvements in treatment are needed, and studying tumor biology has helped to postulate new therapeutic approaches and optimize treatment for better disease outcomes. A fast-track designation by the US FDA to oxaloacetate (OAA) as an "add-on" therapy to standard treatment of care for the treatment of patients with newly diagnosed glioblastoma multiforme (GBM) sparked curiosity to understand its role in gliomas. Hence, a review was carried out to understand the target sites of OAA in the complex metabolic pathway of gliomas.

## Review

Metabolic pathway of normal brain cells and glioma cells: A comparative outlook

Normal human brain cells use about 25% of the body's glucose, with neurons having greater rates of oxidative metabolism and astrocytes depending more on glycolytic pathways [10,11]. A key function of eukaryotic cells is the creation of adenosine triphosphate (ATP) from glucose, which produces either lactate through glycolysis or carbon dioxide and water through oxidative phosphorylation (OXPHOS). Specific glucose transporters (GLUTs) allow cells to absorb glucose, and the first step in the metabolism of glucose is the conversion of glucose to glucose-6-phosphate by hexokinase (HK). The conversion of phosphoenolpyruvate to pyruvate, which is facilitated by pyruvate kinase (PK), is the last stage of glycolysis. The end product of glycolysis, pyruvate, is then able to enter the Krebs (tricarboxylic acid, TCA) cycle during the process of oxidative phosphorylation (OXPHOS). The Krebs cycle is the main source of energy for cells and is a crucial part of aerobic respiration. It consumes pyruvate to produce carbon dioxide, ATP, nicotinamide adenine dinucleotide hydrogen (NADH), and flavin adenine dinucleotide hydrogen (FADH). NADH is then supplied into the electron transport chain, which results in 36 molecules of adenosine triphosphate (ATP) being produced for every glucose molecule. When normal cells are unable to effectively produce ATP by oxidative phosphorylation during hypoxia, glycolysis takes over as the primary source of ATP production. Here, glyceraldehyde 3-phosphate dehydrogenase (GAPDH) utilizes nicotinamide adenine dinucleotide (NAD+) to change glyceraldehyde 3-phosphate (GADP) into 1,3-bisphosphoglycerate (1,3BPG). NAD+ is necessary to make the sixth stage of glycolysis possible. When the oxygen supply is limited, NAD+ is created from NADH by lactate dehydrogenase A (LDHA) in order to continue glycolysis, producing lactate as a byproduct; this process is known as anaerobic glycolysis, yielding two net ATP for every glucose molecule. Normally, NAD+ is regenerated through oxidative phosphorylation by the electron transport chain. Anaerobic glycolysis is 100 times faster than oxidative phosphorylation, despite the fact that it is less effective, allowing it to meet short-term energy needs in the absence of enough oxygen at the cost of a higher glucose consumption [12].

**Figure 1:**
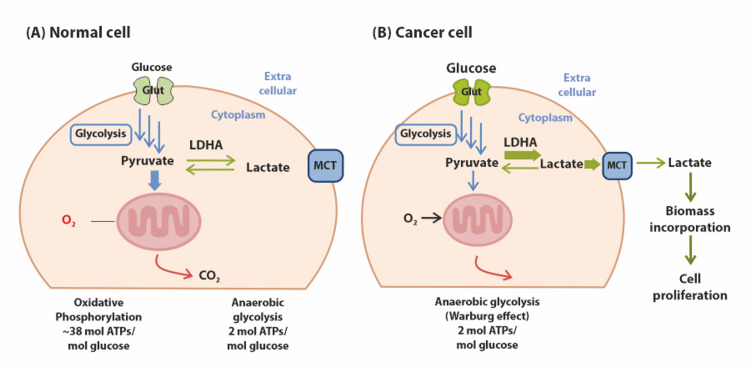
Difference between glycolysis mechanisms in cancer cells and normal cells (A) In the presence of oxygen, normal cells use the glycolysis, TCA cycle, and electron transport system to create up to 38 ATPs of carbon dioxide for every glucose molecule. Pyruvates build up in a hypoxic environment without going through the TCA cycle. The muscle tissue's stored pyruvates are transformed into lactic acid, but only two ATPs are generated as a result (short period). (B) Cancer cells only use the glycolysis process, whether oxygen is present or not; two ATPs are produced from each glucose molecule, and more glucose is needed to provide energy in comparison to normal cells [21] Note: Image sourced from "Regulation of cancer metabolism by deubiquitinating enzymes: the Warburg effect," by Kim SH and Baek KH, 2021, Int J Mol Sci, 22(12), 6173 [21] under Creative Commons Attribution License CC BY 4.0 MCT, monocarboxylate transporter; LDHA, lactate dehydrogenase A; ATPs, adenosine triphosphates, TCA, tricarboxylic acid, O_2_, oxygen; CO_2_, carbon dioxide

Deregulating the normal cellular energy metabolism is now recognized as a fundamental hallmark of cancer, as proposed by Hanahan [13]. Unlike normal cells, cancer cells adopt an altered metabolic pathway, first observed by Warburg in the 1920s [14]. The Warburg effect alters the microenvironment to promote the proliferation of cancer cells by the creation of lactate (L- and D-lactate). In doing so, it fosters increased tumoral cell proliferation, survival, migration, invasion, and angiogenesis, as well as the suppression of the immune system's anticancer response. It also produces a tumoral acidic microenvironment. The generation and conversion of lactate (lactate dehydrogenase {LDH} A, LDHB, LDHD, glyoxalase {GLO} 1, and GLO2), transport (monocarboxylate transporter {MCT} 1 and MCT4), and receptor interaction (G protein-coupled receptor {GPR} 81 and GPR132) all contribute to lactate metabolism in the tumor microenvironment (TME). The alteration is due to a hypoxia condition where the transcription factor hypoxia-inducible factor-1 (HIF-1) is quickly upregulated by cancer cells. HIF-1 is a key regulator of cancer cell metabolism and affects almost every aspect of cancer cell phenotype. A crucial HIF-1 target, lactate dehydrogenase A (LDHA), catalyzes the conversion of pyruvate to lactate and ensures cell survival in hypoxic environments by making up for the loss of oxidative mitochondrial activities. So, it was postulated that it mediates the two-way conversion of pyruvate and lactate. Hence, elevated LDHA concentrations are a common feature of tumors, including glioma cells, and are associated with a poor prognosis [15-22]. Therefore, the Warburg effect, the lack of oxygen-induced glycolysis inhibition in cancer cells, is a phenomenon of great interest. Figure 1 shows the difference between glycolysis mechanisms in cancer cell "Warburg effect" and normal cells.

The proposed theory of OAA action on altering the Warburg effect

**Figure 2:**
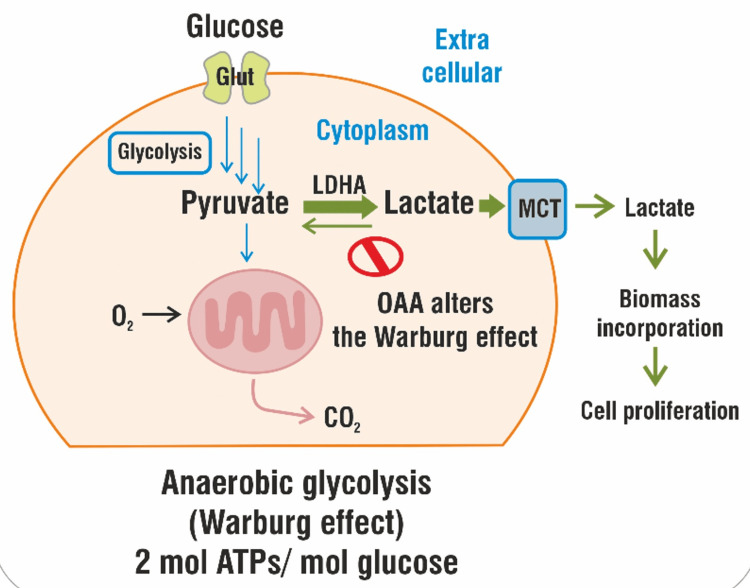
Oxaloacetate (OAA) alters the "Warburg effect" OAA alters the "Warburg effect" by reducing the concentration of LDHA concentration in gliomas [22] Note: Image sourced from “Enzymatic activation of pyruvate kinase increases cytosolic oxaloacetate to inhibit the Warburg effect,” by Wiese EK, Hitosugi S, Loa ST, et al., 2021, Nat Metab, 3(7), 954-68 [22] under Creative Commons Attribution License CC BY 4.0 MCT, monocarboxylate transporter; LDHA, lactate dehydrogenase A; ATPs, adenosine triphosphates; O_2_, oxygen; CO_2_, carbon dioxide

In cancer cells, oxaloacetate may have a direct impact on the Warburg effect. Human lactate dehydrogenase A (LDHA) is competitively inhibited by oxaloacetate (OAA), which prevents LDHA from functioning in cancer cells. OAA fosters an advantageous (alkaline) cellular environment that allows for the efficacy of other treatments. Figure 2 shows the proposed mechanism of OAA for reducing LDHA in cancer cells, thereby reversing the Warburg effect.

Evidence of the proposed theory

A study investigated the impact of oxaloacetate (OAA) supplementation on cancer cell energy metabolism. This study investigated whether oxaloacetate may affect altered cellular metabolism in cancer cells, particularly glioblastoma multiforme. They used 13C isotopomer analysis based on gas chromatography-mass spectrometry (GC-MS) to look at how glucose is used differently in GBM cells taken from different patients. The cells were cultured in Dulbecco's Modified Eagle Medium (DMEM) media supplemented with 2 mM oxaloacetate and glucose for 10 days. The addition of [U-13C]glucose six hours before harvesting reduced 13C labelling by 19.7% and 48.8% in the pyruvate and lactate pools, respectively, compared to cells not treated with OAA for 10 days. Warburg glycolysis was drastically reduced with OAA supplementation and has been found to improve neuronal cell bioenergetics and trigger brain mitochondrial biogenesis [23].

Theory of glutamine scavenging

The Warburg effect is a major metabolic characteristic of cancer. Proliferating cells require not only ATP but also other cellular components. Glutamine metabolism provides nitrogen and carbon for the biosynthesis of nucleotides and amino acids. It can also replenish the carbon backbone by serving as an anaplerotic substrate for the tricarboxylic acid (TCA) cycle. Additionally, the metabolism of glutamine helps produce NADPH for the synthesis of fatty acids. Glutaminase (GLS) initially converts glutamine to glutamate during glutaminolysis. For the purpose of preserving cellular homeostasis, glutamate dehydrogenase (GDH) can metabolize glutamate that is obtained from glutamine to form alpha-ketoglutarate (alpha-KG). The astrocytic enzyme glutamine synthetase (GS) can also convert glutamate to glutamine. Increased glutamine uptake within tumors has been seen in human gliomas but not in healthy brains, and transformed cells exhibit a high rate of glutamine metabolism during fast proliferation. The transaminase inhibitor aminooxyacetate (AOA) has been shown to cause selective toxicity in MYC-transformed cells. It has been shown that MYC-dependent metabolic change, or glutaminolysis, renders cells hooked to glutamine for protein and nucleotide production. GS maintains de novo purine production in glutamine-deficient GBM cells [24-28]. Additionally, pharmacological resistance to mammalian target of rapamycin (mTOR) kinase inhibitors is facilitated by glutamine metabolism in a KG-dependent way. The compensatory elevation of GLS and glutamate confers advantages in terms of survival. The cysteine/glutamate antiporter (xCT), expressed by the *SLC7A11* gene, enhances glutamate secretion and cystine absorption when mTOR kinase is inhibited. Glioma cells depend on cysteine absorption to maintain cellular redox equilibrium. mTOR complex 2 (mTORC2) selectively binds to xCT and phosphorylates it on serine 26, decreasing its activity. xCT causes glioma-mediated neuronal damage through the release of glutamate [29-32].

Proposed theory of OAA action on glutamine scavenging

**Figure 3:**
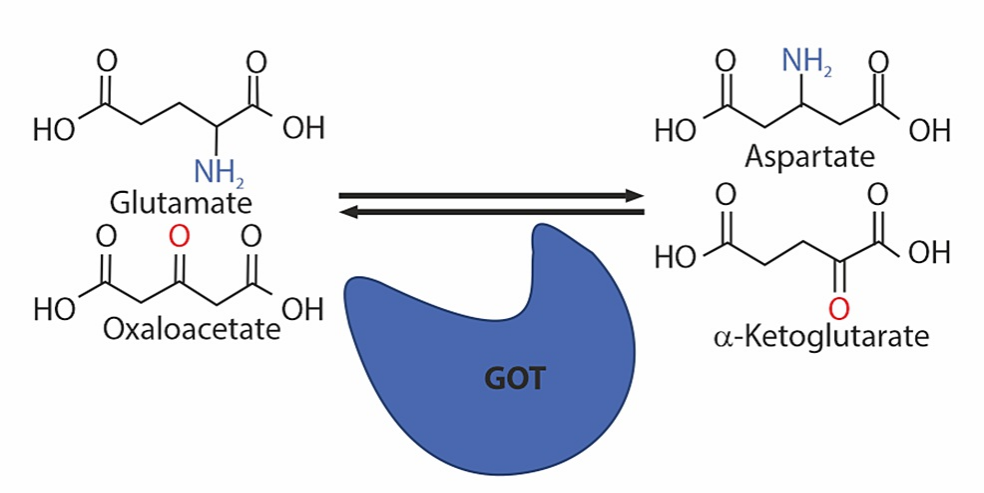
Fundamental of glutamate scavenging Glutamate-oxaloacetate transaminase (GOT) transforms glutamate and oxaloacetate into aspartate and alpha-ketoglutarate by transferring the amino group from glutamate to oxaloacetate and replacing the amino group of glutamate with a carbonyl group [34] Note: Image sourced from "Glutamate scavenging as a neuroreparative strategy in ischemic stroke," by Kaplan-Arabaci O, Acari A, Ciftci P, and Gozuacik D, 2022, Front Pharmacol, 13, 866738 [34] under Creative Commons Attribution License CC BY 4.0

Oxaloacetate can contribute to reducing gliomas by glutamate scavenging and promoting the conversion of glutamate to alpha-ketoglutarate in the TCA cycle, also reducing NADPH levels. Glutamate-oxaloacetate transaminase (GOT) turns glutamate and oxaloacetate into aspartate and alpha-ketoglutarate by transferring the amino group from glutamate to oxaloacetate while replacing the amino group of glutamate with a carbonyl group (Figure 3). This fundamental principle disrupts the process of glutamine synthesis, which is essential for cell growth and proliferation. Oxaloacetate's propensity to direct glutamine-derived carbon away from NADPH synthesis pathways can lead to decreased cell viability. This can result in cell death and apoptosis, which inhibits the formation of gliomas. Inhibiting glutaminolysis can reduce the quantity of glutamate produced, further inhibiting the growth of glioma cells [33,34].

Evidence of the proposed theory

A preclinical study demonstrated that blood L-glutamine scavenging offers neuroprotection against gliomas. The study involved brain-implanted gliomas in rats and mice and administered oxaloacetate or human GOT (hGOT) injections or a combination of both. Oxaloacetate-consuming mice had lower tumor volumes, less invasive behavior, and longer life spans compared to saline-drinking control mice [35]. Another preclinical study on glioblastoma multiforme found that daily human equivalent doses of oxaloacetate, given in various dosages, significantly increased survival in mice. The study also found that temozolomide, a combination of oxaloacetate and temozolomide, also showed a significant increase in survival compared to the vehicle control. The time to endpoint (TTE) for each mouse was recorded, and the log-rank test was used to assess the differences between the two groups' survival experiences. The study concluded that oxaloacetate and temozolomide did not display any unfavorable side effects at the doses investigated [36].

## Conclusions

Glioma, an aggressive brain cancer, presents a significant challenge in oncology due to its rapid progression and resistance to conventional therapies. Recent research has concentrated on comprehending the metabolic reprogramming in cancer cells, including glioma. Oxaloacetate (OAA) has emerged as a promising molecule due to its potential to disrupt the Warburg effect and hinder glioma progression when used in adjunct with standard chemotherapy, offering a multifaceted approach for therapeutic intervention.

Furthermore, OAA's potential to enhance oxidative phosphorylation, restore normal TCA cycle activity, and promote efficient utilization of mitochondrial metabolism may ultimately shift the metabolic balance within glioma cells toward a less aggressive phenotype. When combined with standard chemotherapy regimens, this emerging therapeutic approach holds promise for achieving substantial improvements in glioma treatment outcomes, providing new hope for patients facing this devastating disease. Further preclinical and clinical investigations are warranted to validate the potential of OAA as a glioma treatment strategy, and its translation to the clinical setting represents an exciting avenue for future research and therapeutic development.
